# Nutrient L-Alanine-Induced Germination of *Bacillus* Improves Proliferation of Spores and Exerts Probiotic Effects *in vitro* and *in vivo*

**DOI:** 10.3389/fmicb.2021.796158

**Published:** 2021-12-02

**Authors:** Shuang Lu, Xianyin Liao, Li Zhang, Ying Fang, Meixian Xiang, Xiaohua Guo

**Affiliations:** ^1^College of Life Science, South-Central University for Nationalities, Wuhan, China; ^2^School of Pharmaceutical Sciences, South-Central University for Nationalities, Wuhan, China

**Keywords:** *Bacillus*, probiotics, L-alanine, spores, germination

## Abstract

As alternatives to antibiotics in feed, probiotic *Bacillus* carries multiple advantages in animal production. Spores undergo strain-related germination in the gastrointestinal tract, but it is still unknown whether the probiotic function of the *Bacillus* depends on the germination of spores *in vivo*. In this study, based on 14 potential probiotic *Bacillus* strains from fermented food and feed, we detected the germination response of these *Bacillus* spores in relation to different germinating agents. The results showed the germination response was strain-specific and germinant-related, and nutrient germinant L-alanine significantly promoted the growth of strains with germination potential. Two strains of *Bacillus subtilis*, S-2 and 312, with or without a high spore germination response to L-alanine, were selected to study their morphological and genic differences induced by L-alanine through transmission electron microscopy and comparative transcriptomics analysis. Consequently, after L-alanine treatment, the gray phase was largely increased under microscopy, and the expression of the germination response genes was significantly up-regulated in the *B. subtilis* S-2 spores compared to the *B. subtilis* 312 spores (*p* < 0.05). The protective effect of L-alanine-induced spore germination of the two strains was comparatively investigated both in the IPEC-J2 cell model and a Sprague–Dawley (SD) rat model challenged by enterotoxigenic *Escherichia coli* K99. The result indicated that L-alanine helped *B. subtilis* S-2 spores, but not 312 spores, to decrease inflammatory factors (IL-6, IL-8, IL-1 β, TNF-α; *p* < 0.05) and promote the expression of occludin in IPEC-J2 cells. Besides, supplement with L-alanine-treated *B. subtilis* S-2 spores significantly improved the growth of the SD rats, alleviated histopathological GIT lesions, and improved the ratio of jejunal villus length to crypt depth in comparison to the *B. subtilis* S-2 spores alone (*p* < 0.05). Improved species diversity and abundance of fecal microbiota were only observed in the group with L-alanine-treated S-2 spores (*p* < 0.05). The study demonstrates L-alanine works well as a probiotic *Bacillus* adjuvant in improving intestinal health, and it also provides a solution for the practical and accurate regulation of their use as antibiotic alternatives in animal production.

## Introduction

Antimicrobial resistance has been recognized as one of the top three major threats to public health by The World Health Organization (Xiong et al., 2018). The antimicrobials applied in feed animals promote the emergence and spread of antimicrobial resistance (Gil-Gil et al., 2019). Several countries have restricted or banned the use of antimicrobials in feed. As alternatives to antibiotics, probiotics have been widely used in feed industries for preventing infections by alleviating antimicrobial-mediated resistance (Ouwehand et al., 2016; Mingmongkolchai and Panbangred, 2018; Abd El-Hack et al., 2020). Particularly, as one of the general sources of probiotics, the spore-forming *Bacillus* species offer several advantages, such as heat stability and resistance to adverse environments of low pH and bile salt toxicity, in comparison to non-spore-formers (Davies, 2010; Setlow, 2010). Spore formation increases the survival of living cells during the manufacture and storage processes, including freezing, drying, thawing, and granulation. Additionally, spores have a stronger ability to survive passage through the stomach and to proliferate in the digestive tract (Casula and Cutting, 2002). Therefore, the *Bacillus* probiotics seemed to be more suitable for application in the feed industry because of their processing and storage stabilities and low production costs.

The efficacy of *Bacillus* species as probiotics has been demonstrated in the control of pathogens (Zhang et al., 2017; Rajabi et al., 2020; Soto, 2021), improvement of intestinal homeostasis (Fujiya et al., 2007; Kang et al., 2021), and anti-oxidant and immune-modulatory abilities in animals (Du et al., 2018; Jing et al., 2018; Mirk et al., 2021). However, in comparison to non-spore-formers, *Bacillus* species as probiotics have been found to have a complexity in cell morphology, including spores and vegetative cells, which are modulated by sprouting and sporulation factors (Tam et al., 2006; Bernardeau et al., 2017). Due to the anaerobic or slightly aerobic environment of the gastrointestinal tract (GIT), the dynamics of germination and sporulation in the GIT and the corresponding mechanism of *Bacillus* spores on the intestinal homeostasis are not easily explored since various pathways are involved in the behaviors (Leser et al., 2010; Bernardeau et al., 2017).

Vegetative cells and spores might show a different effect on the protective activities *in vitro* and *in vivo*. Treatment with *Bacillus megaterium* SF185 spores has been found to prevent or reduce the damages caused by oxidative stress both at the level of Caco-2 cells and in a dextran sodium sulfate-induced mouse model (Mazzoli et al., 2019). *Bacillus* SC06 vegetative cells alleviated oxidative stress-induced disorders and apoptosis *via* p38-mediated autophagy in rat jejunum and IEC-6 cells (Wu et al., 2019). Bs 29784 vegetative cells were found to significantly reduce the upregulation of iNOS protein levels in Caco-2 cells (Rhayat et al., 2019). The vegetative variants of Bs PB6-PR induced higher levels of IL-10, TNF-α, and IFN-γ than prepared spore powder (Selvam et al., 2009). Huang et al. showed that the vegetative cells of *Bacillus subtilis* could stimulate expression of the Toll-like receptors (TLR) genes containing TLR2 and TLR4, whereas spores could not (Huang et al., 2008). In some comparative studies, the *Bacillus* strain of Bs 29784, but not Bs A and Bs B, was able to specifically decrease IL-8 production and increase the transepithelial electrical resistance of differentiated Caco-2 cells, and *Bacillus* SC06 showed more significant intestinal tissue repair and antioxidant properties than SC08 in a rat model (Rhayat et al., 2019; Wu et al., 2019). Therefore, the protective effect of *Bacillus* might be strain-specific and cell type-related.

Moreover, the activity of postbiotics is closely associated with the mode of action of probiotic *Bacillus*. The International Scientific Association of Probiotics and Prebiotics has defined postbiotics as the “preparation of inanimate microorganisms and/or their components that confers a health benefit on the host” (Salminen et al., 2021). Some antimicrobial substances produced by *Bacillus*, including subtilin, coagulin, surfactin, and bacilysin, were considered one of the main mechanisms of pathogen exclusion in the GIT (Urdaci et al., 2004). Piewngam et al. demonstrated that a *Bacillus* lipopeptide, fengycin, restricted intestinal *Staphylococcus aureus* colonization by inhibiting quorum sensing (Piewngam et al., 2018). The *B. subtilis*-derived competence and sporulation factor, as a quorum-sensing pentapeptide, activated the Akt and p38 MAPK pathways and protected epithelial cells from oxidant stress in the intestine (Di Luccia et al., 2016).

When living *Bacillus* cells are ingested into the intestine in the form of spores, the spores usually undergo a life cycle consisting of germination, growth, and re-sporulation, which is responsible for the metabolic and immunomodulatory mechanisms in *Bacillus* (Tam et al., 2006; Bernardeau et al., 2017). Studies on the *Bacillus* germination dynamics were largely conducted *in vitro,* and the germination of *Bacillus* spores was strain-specifically triggered by both nutrient and non-nutrient germinants (Real et al., 2005; Lovdal et al., 2012; Amon et al., 2020; Christie and Setlow, 2020). Moreover, *Bacillus* germination and sporulation processes in the GIT have also been observed in many studies in broilers, pigs, human beings, and mice (Jadamus et al., 2001; Casula and Cutting, 2002; Leser et al., 2010; Ghelardi et al., 2015). However, to the best of our knowledge, the association between the selection of *Bacillus* strains and germination potential *in vitro* and the corresponding protective effect *in vivo* has not yet been investigated. Nutrient germinants were hypothesized to induce the spore germination of a *Bacillus* strain in the GIT and improve the metabolic activity and the protective effect on intestinal homeostasis. Thus, the present study aimed to identify and characterize *Bacillus* spores with germination potential and to determine whether the germination was improved by L-alanine as a germinant to stimulate the protective efficacy in IPEC-J2 cells and rats challenged by *Escherichia coli* (ETEC) K99.

## Materials and Methods

### Bacterial Strains

Fourteen wild-type *Bacillus* strains were originally isolated from fermented food and feed. They were screened for potential spore-forming probiotics based on the functionalities, safety, and stress resistance reported by Mingmongkolchai and Panbangred (2018). All the strains were identified according to morphology, physiological and biochemical tests, and sequencing of 16S rDNA. *B. subtilis* 168 was used as the standard model strain from *Bacillus* species, and it is also used as a reference strain to compare the difference in germination response of *Bacillus* from other strains (Moir et al., 1991; Alzahrani and Moir, 2014). The evolutionary trees (Supplementary Figure 1) of the strains involved in the study were drawn according to the 16S rDNA sequences of each isolated strain. The ETEC K99 was purchased from the China Center of Veterinary Culture Collection (CVCC; C83529). The ETEC K99 was activated and cultured in nutrient broth. All the strains used in the study were stored in 20% sterile glycerol at −80°C until needed.

### Preparation of the Spores

The *Bacillus* strains were grown and sporulated in Difco sporulation medium (DSM) from 48 h to 60 h at 37°C. The spores were harvested after a heat shock of 80°C for 15 min. The spores were washed twice and then resuspended in phosphate-buffered saline (PBS) buffer. Each spore suspension was sampled immediately to determine the number of colony-forming units (CFUs) per milliliter before use. The spore concentration was adjusted depending on the requirements of each experiment. The spores were freshly prepared for each experiment.

### *Bacillus* Spore Germination Assay

Eight nutrient mediators that might be present in the intestine were used as the germination agents, including D-glucose, L-alanine, L-glutamine, L-aspartate, L-valine, L-lysine, L-glutamate, and complex sprouting agents AGFK (100 mmL-aspartate + 10 mm D-glucose + 10 mm D-fructose + 10 mm KCl) based on the report of Yi and Setlow (2010). Each germination agent was dissolved in 50 mm Tris–HCl buffer (pH 8) and prepared to a concentration of 100 mm. Freshly prepared spore suspensions were diluted by Tris–HCl buffer to a concentration of approximately 10^7^ CFU/ml. Then, the dilution was treated immediately to check the germination potential based on the release of dipicolinic acid (DPA) from the endospores. Two methods used were for heat shock and germinant–triggered germination based on the method of Liang et al. (2018). For the heat shock, the spore suspensions were autoclaved in screw-cap glass test tubes at 121°C for 10 min for the full release of DPA. After cooling, the supernatants containing the DPA were sampled after centrifugation (2,500 × *g* for 10 min) and tested for fluorescence intensity based on the method of Liang et al. (2018). Briefly, the supernatants were mixed with EuCl_3_ (2 mm) and 1,2-cyclohexanediamine-N,N,N′N′-tetraacetic acid (CyDTA; 2 mm) in the proportion of 1:4.5:4.5 by a vortex oscillator. The fluorescence intensity of the complexes were quantified by a Hitachi F-7000 spectrofluorophotometer (Hitachi Ltd., Tokyo, Japan; 272 nm excitation and 619 nm emission) with the pre-set parameters of a 5 nm/10 nm slit and a photo-multiplier tube voltage of 700V. The fluorescence intensity of the supernatants treated by heat shock was designated as AU1.

In the germinant–treated method, the freshly prepared spores were centrifuged and resuspended in 100 mm germinant solution buffered by 50 mm Tris–HCl. The fluorescence intensity of the germinant–treated supernatants were tested as AU2. The germination potential of each spore treated by different germinants was evaluated based on the relative fluorescence intensity expressed by AU2/AU1. The time and concentration effects on the germination of the spores treated by L-alanine as a universal germinant were studied by setting specific L-alanine concentration gradients and time sampling gradients, then testing the relative fluorescence intensity.

### Spore Growth Assay

One strain from each *Bacillus* species (*B. subtilis*, *Bacillus cereus*, *Bacillus licheniformis*, and *Bacillus coagulans*) with significant germination potential was selected for the spore growth assay. One strain of *B. subtilis* without germination response to 100 mm L-alanine was included as the control. The synthetic medium (10 g/L glucose, 10 g/L urea, 5 g/L diamine citrate, 1.5 g/L KH_2_PO_4_, 1.5 g/L NaNO_3_; pH 7.0) in a 50 ml/250 ml Erlenmeyer flask was used to exclude the possible interference of nutrient germinants. For each strain, two experimental treatments were set with three replications, including a spore-treated group and an L-alanine-pretreated group. In the spore-treated group, 200 μl of the spore suspensions with the initial concentration of 10^8^ CFU/ml was directly inoculated into the 50 ml synthetic medium. In the L-alanine-pretreated group, the 10^8^ CFU/ml spore suspensions were centrifuged at 12,000 rpm for 3 min, and the pellets were resuspended in Tris–HCl buffer containing 100 mm L-alanine. After 30 min of incubation at 37°C, the bacteria were collected, washed twice, and resuspended in PBS buffer in an equal volume. Then, 200 μl of the L-alanine pretreated-spore suspensions were inoculated into the 50 ml synthetic medium. The culture conditions were set on a rotating shaker at 37°C and 200 rpm. The samples were selected at 3 h intervals for the detection of cell optical density at 600 nm.

### Transmission Electron Microscopy

To monitor the germination in the spores treated by L-alanine, purified spores were concentrated and then resuspended in Tris–HCl buffer or Tris–HCl buffer containing 100 mm L-alanine respectively, followed by incubation with agitation at 37°C for 30 min. The collected cells were fixed in 2.5% glutaraldehyde for 4 h at 4°C and post-fixed with 1% osmium tetroxide in PBS (pH 7.4) for 2 h at 20°C. The samples were washed three times with PBS and then dehydrated with graded series of alcohol (50, 70, 80, 90, 95, 100% for 15 min, respectively). Subsequently, the samples were embedded into Epon that were polymerized at 65°C for more than 48 h. Finally, the samples were cut into 60–80 nm sections and stained with uranyl acetate, and counterstained with lead citrate. The Transmission electron microscopy (TEM) images were captured using a transmission electron microscope (Hitachi, HT7800/HT7700).

### Comparative Transcriptomics Analysis

Based on the results of the spore germination assay and growth assay, two strains of *B. subtilis*, S-2 and 312, were included in the comparative transcriptomics analysis. The spores of *B. subtilis* S-2 were selected because of their high germination potential to L-alanine. The spores of *B. subtilis* 312 without a response to L-alanine were used as the control. Similar to the treatments in the spore growth assay, the two strains were inoculated into the synthetic medium according to the spore-treated group and L-alanine-pretreated group, respectively. The cells with or without L-alanine pretreatment were cultured in the synthetic medium for 9h, and then collected. The pellets were washed three times with PBS and resuspended in 100 μl of lysozyme (10 mg/ml) in a water bath at 37°C for 30 min. Then, 400 ml of TRIzol reagent (Invitrogen Life Technologies) was added, and total RNA was extracted according to the instructions provided by the manufacturer. The quality of the total RNA was assessed by NanoDrop 2000 (Thermo Scientific) and Bioanalyzer system (Agilent). Then ribosomal RNA was removed using the Zymo-Seq RiboFree Total RNA Library Kit (Irvine, CA, United States). The RNA was then fragmented to 200–300bp, followed by random primers and reverse transcriptase to synthesize the first strand cDNA and then the second strand of cDNA. The fragments were purified using the AMPure XP beads (Beckman Coulter, Beverly, CA, United States) to enrich cDNA of 400–500bp. The library was then quantified by the Agilent high sensitivity DNA assay and finally sequenced on Hiseq X ten platform (Illumina).

The raw data were filtered according to the following criteria: (1) Cutadapt (v1.15) is used to remove the adaptor sequence at the 3′ end; (2) the reads are removed if their average quality score is lower than Q20. Then, the filtered reads are mapped to the reference genomes^1^ using the Bowtie2 tool (v2.2.6).^2^ According to the sequence alignment results, the expression level of each gene was calculated, and the expression level was normalized by Fragments Per Kilo bases Per Million Fragments (FPKM). DESeq (version 1.18.0) was used to identify the differentially expressed genes (DEGs). The DEGs were selected with |log_2_(fold change)|>1 and a value of *p* < 0.05. All genes were mapped to terms in the Gene Ontology (GO) database and calculated the numbers of DEGs in each Term. Using topGO to perform GO enrichment analysis and ClusterProfiler (3.4.4) software carry out the enrichment analysis of the KEGG pathway on the differential genes. A value of *p* < 0.05 was considered statistically significant.

### Co-culture of IPEC-J2 With Spores and ETEC Infection

The IPEC-J2 cells were purchased from the China Center for Type Culture Collection and were cultured in T25 flasks with Dulbecco’s modified Eagle’s medium (DMEM) with 10% fetal bovine serum (Gibco) and 1% penicillin–streptomycin solution at 37°C in 5% CO_2_. The IPEC-J2 cells were plated on 24- or 6-well plates at a density of 10^5^ cells/cm^2^ before the experiments. Spores of *B. subtilis* S-2 and 312 were pretreated with PBS or PBS containing L-alanine (100 mm) for 2 h at 37°C, and then they were washed three times with PBS. The IPEC-J2 cells were incubated with PBS or L-alanine pretreated *B. subtilis* S-2 and 312 spores (10^7^ CFU/ml) for 16 h. Then, the cells were infected with ETEC (10^6^ CFU/ml) for another 12 h. Finally, the cells were harvested for quantitative reverse transcription polymerase chain reaction (RT-qPCR) and Western blot (WB) analysis.

### RT-qPCR Analysis of the Inflammatory Cytokines

Total RNA of the IPEC-J2 cells was isolated with TRIzol reagent (Invitrogen), following the manufacturer’s instructions. The mRNAs were reverse transcribed using reverse transcriptase (HiScript II Q Select RT SuperMix for qPCR, Vazyme, Nanjing, China). qPCR was performed with SYBR Green qPCR Master Mix (qPCR SYBR Green Master Mix, Vazyme). GAPDH was used as a housekeeping gene. The expression levels of the inflammatory cytokines, IL-6, IL-8, IL-1 β, and TNF-α, were calculated based on the change-in-cycling-threshold (2^−∆∆Ct^) method. The primers used in qPCR are shown in Table 1.

**Table 1:** Primers for RT-qPCR.

Primers	Sequence (5'→3')
GAPDH-F	TGGTGAAGGTCGGAGTGAAC
GAPDH-R	GGAAGATGGTGATGGGATTTC
IL-1 β-F	GGCCATAGTACCTGAACCCG
IL-1 β-R	CCAAGGTCCAGGTTTTGGGT
IL-6-F	TGGCAGAAAAAGACGGATGC
IL-6-R	TACTAATCTGCACGGCCTCG
IL-8-F	GCCTTCTTGGCAGTTTTCCTG
IL-8-R	TGGAAAGGTGTGGAATGCGTA
TNF-α-F	CAACGGCGTGAAGCTGAAAG
TNF-α-R	AGACCCCTCCCAGGTAGATG

### WB Analysis of the Tight Junction Proteins and IL-6

The IPEC-J2 cells were first lysed with RIPA lysis buffer (Servicebio, Wuhan, China, G2002) for 30 min on ice, and the supernatant was collected after centrifugation. The protein concentration was determined by bicinchoninic acid assay (Servicebio, Protein Quantification Kit G2026). A total of 40 μg of protein mixed with 5 × SDS loading buffer was electrophoresed in an 8–12% sodium dodecyl sulfate polyacrylamide gel electrophoresis and transferred to a polyvinylidene fluoride membrane. The membrane with protein blots was first blocked in tris-buffered saline with 0.1% Tween-20 containing 5% bull serum albumin, then incubated with the primary antibody at 4°C overnight, and finally incubated with horseradish peroxidase (HRP)-labeled secondary antibody at room temperature for 1h, and later washed five times. Finally, the pictures were taken by a Gel Imager System (Bio-Rad, United States). The antibodies used in this study are listed in Table 2.

**Table 2:** The antibodies used in WB and IFA analysis.

Antibodies	Source	Identifier
Claudin1 rabbit polyclonal antibody	protrintech	Cat#13050-1-AP
Occludin mouse polyclonal antibody	protrintech	Cat#66378-1-lg
IL6 rabbit pAb	ABclonal technology	Cat#A0286
GAPDH mouse mAb	ABclonal technology	Cat#AC002
HRP goat anti-mouse IG(H + L)	ABclonal technology	Cat#AS003
Anti-rabbit IgG HRP-linked antibody	Cell signaling technology	Cat#7074S
DAPI	Beyotime	Cat#C1005

### Animal Experiments

Thirty-six weaned male Sprague–Dawley (SD) rats were randomly divided into six treatment groups with six rats per treatment. After 3 days of acclimation, the rats underwent six experimental treatments as follows: (1) the CON group: daily oral administration of PBS; (2) the ETEC group: daily oral administration of PBS for 14 days, followed by ETEC K99 oral gavage challenge; (3) the S-2 + ETEC group: daily oral administration of *B. subtilis* S-2 spore suspensions for 14 days, followed by ETEC K99 gavage challenge; (4) the S-2 + L-alanine + ETEC group: daily oral administration of L-alanine-pretreated *B. subtilis* S-2 spore suspensions for 14 days, followed by ETEC K99 challenge; (5) the 312 + ETEC group: daily oral administration of *B. subtilis* 312 spore suspensions for 14 days, followed by ETEC K99 challenge; and (6) the 312 + L-alanine + ETEC group: daily oral administration of L-alanine-pretreated *B. subtilis* 312 spore suspensions for 14 days, followed by ETEC K99 challenge. The inoculation concentration of *Bacillus* was adjusted to 10^7^ CFU/ml in the PBS drinking water. In the challenge groups, all the rats were intragastrically inoculated with 1 ml dose of PBS solution containing 10^8^ CFU/ml of ETEC K99 on day 15. Individual body weight and water intake were recorded daily for the duration of the study. The rats were sacrificed by cervical vertebra dislocation on day 18 to collect the jejunum samples and fresh feces in the anus.

### Hematoxylin & Eosin Staining

The rats’ jejunum was fixed with 4% paraformaldehyde and embedded with paraffin. After cutting the tissues into 5- μm sections, they were mounted on a glass slide and deparaffinized in xylene, and rehydrated in a graded ethanol series. The tissue sections were washed in distilled water for 2 min, incubated with hematoxylin solution at 37°C for 5 min, immersed five times in a solution of 1% HCl and 70% ethanol, and subsequently washed with distilled water for 10 min. The sections were then incubated with eosin solution at 37°C for 2 min, dehydrated with alcohol, and immersed in xylene. Finally, the Hematoxylin & eosin (H&E)-stained slides were mounted with neutral gum and then covered with a coverslip for viewing with a microscope.

### Immunofluorescence Assays

The rat jejunum sections underwent H&E staining, were incubated with EDTA solution (pH 9.0), and were heated in a microwave for antigen retrieval. After heat treatment, the slides were cooled to room temperature and washed three times in PBS. The sections were subsequently blocked with 3% BSA for 40 min at room temperature and incubated with mouse anti-occludin antibody at 4°C overnight. The sections were then washed three times with PBS, followed by incubation with Dylight 594-conjugated anti-mouse IgG (Abbkine) for 1 h at RT. After washing three times, the nuclei were stained with DAPI for 15 min, and the cells were imaged by fluorescence microscopy (Nikon Eclipse C1, Japan).

### 16S rRNA Sequencing of Microbiota and Data Analysis

Total genomic DNA of the feces was extracted using a PowerSoil^®^ DNA Isolation kit (MoBio Laboratories, Carlsbad, CA, United States) following the manufacturer’s instructions. DNA quantity and quality were evaluated by NanoDrop 2000 (Thermo Scientific, United States) and the A260/A280 ratio. Phusion (New England Biolabs) and primers MPRK341F (5'-ACTCCTACGGGAGGCAGCAG-3') and MPRK806R: (5'-GGACTACHVGGGT WTCTAAT-3') targeted the V3 + V4 region of the 16S rRNA gene and were used for PCR. The PCR products were purified using gel electrophoresis followed by the MinElute^®^ PCR Purification Kit (QIAGEN, Hilden, Germany) and VAHTSTM DNA Clean Beads (Vazyme, Nanjing, China). The libraries were constructed using the TruSeq Nano DNA LT Library Prep Kit (Illumina) and sequenced using HiSeq2500 (Illumina). The raw reads were merged and filtered into clean reads using Quantitative Insights into Microbial Ecology (QIIME) version 1.9.1 (Caporaso et al., 2010). The operational taxonomic units (OTUs) with ≥97% sequence similarity were annotated using the SILVA database (bacteria, http://www.arb-silva.de; Quast et al., 2013). Each OTU was generally considered to be a microbial species. OTU analysis was used to calculate the microbial diversity and abundance of the different samples. The linear discriminate analysis (LDA) effect size (LEfSe) was performed to determine the differences in abundance; the threshold of the LDA score was 4.0.

### Statistical Analyses

Statistical analysis was performed using GraphPad Prism software (version 7.0). Data were expressed as means ± standard deviation (SD), and statistical differences were determined using one-way ANOVA. The value of *p* < 0.05 was considered statistically significant. All the experiments were repeated at least three times.

## Results

### Response of *Bacillus* Strains to Different Nutrient Germinants

**Figure 1:**
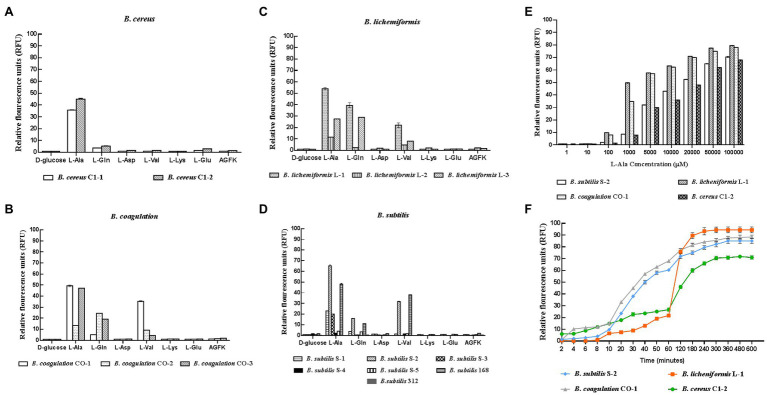
Response of different probiotic *Bacillus* strains to different nutrient germinants. D-glucose, L-alanine (L-Ala), L-glutamine (L-Gln), L-aspartate (L-Asp), L-valine (L-Val), L-lysine (L-Lys), L-glutamate (L-Glu), and complex sprouting agents AGFK. **(A–D)** *Bacillus cereus*, *Bacillus coagulans*, *Bacillus lichemiformis*, *Bacillus subtilis*, respectively. **(E)** Concentration effect of L-alanine on DPA release of spores. **(F)** Time effect of L-alanine treatment on DPA release of spores.

In the 14 *Bacillus* strains isolated for the potential spore-former probiotics, plus the standard strain of *B. subtilis* 168, the result presented in Figures 1A–D showed that the germination response was strain-specific and germinant-related. Most of the spores from the different *Bacillus* species were sensitive to L-alanine, L-valine, and L-glutamine. In the present study, L-alanine showed the potential to be a general germinating agent, which could induce the release of DPA and trigger the germination effect on most *Bacillus* spores. However, in the *B. subtilis* species, L-alanine greatly increased the release of DPA of *B. subtilis* S-2 (66.06%); and almost no release was seen in *B. subtilis* 312 (1.97%; Figure 1D). The strains from each species with higher germination potential were selected to study the time effect and concentration-effect exerted by L-alanine. As seen in Figures 1E,F, the germination of the spores was associated with the L-alanine concentration and treatment time. The spore germination was triggered by at least 100 μm of L-alanine, and the maximum release of DPA could be reached at 100 mm L-alanine treatment for at least 3 h.

### The Effect of L-Alanine on the Growth of *Bacillus*

**Figure 2:**
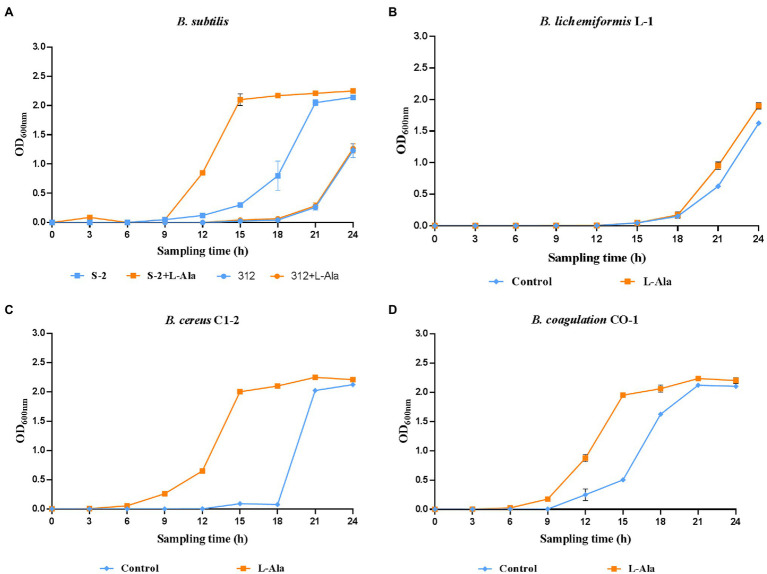
The growth curves of **(A)** *B. subtilis* S-2/312, **(B)** *B. lichemiformis* L-1, **(C)** *B. cereus* C1-2, **(D)** *B. coagulans* CO-1 with or without L-alanine pre-treatment.

The strains in each *Bacillus* genus that were the most responsive to L-alanine were selected, and inactive *B. subtilis* 312 was used as the control. The growth curves show that L-alanine significantly promoted the growth of spores in all the *Bacillus* strains in the synthetic medium, except for *B. subtilis* 312, compared to the untreated spores (Figures 2A–D). In the same *Bacillus* genus, *B. subtilis* S-2 and *B. subtilis* 312 showed a great discrepancy in germination in response to L-alanine as well as differences in the growth-promoting effect (Figure 2A). Therefore, their biological characteristics and functions affected by L-alanine were further explored and compared in the subsequent experiments.

### Different TEM-Based Morphology of *B. subtilis* S-2 and 312 Treated by L-Alanine

**Figure 3:**
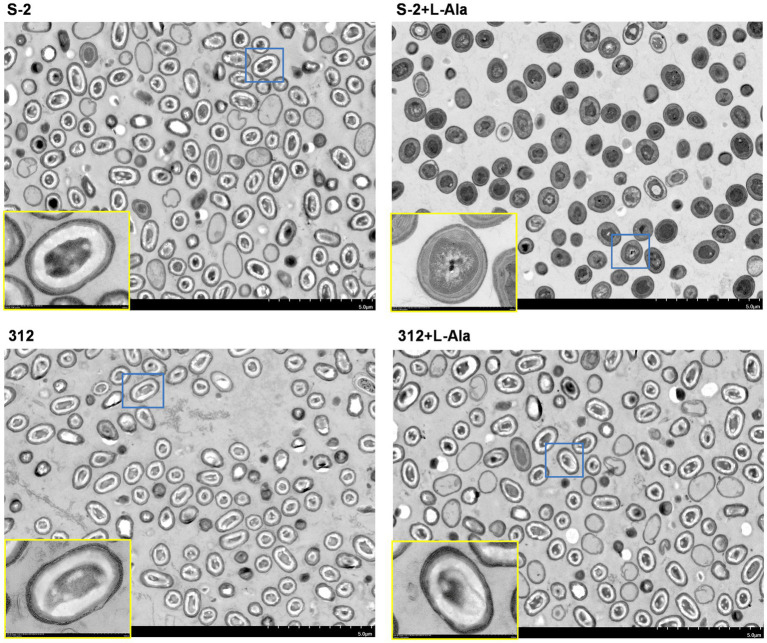
Morphological changes of *B. subtilis* S-2 and 312 with L-alanine treatment. The cell morphology of *B. subtilis* S-2 spores, L-alanine treated *B. subtilis* S-2 spores, *B. subtilis* 312 spores, L-alanine treated *B. subtilis* 312 spores were displayed by transmission electron microscopy (TEM). The yellow frame is an enlarged version of the blue frame.

The differential morphological changes that occurred after L-alanine treatment were observed by TEM based on *B. subtilis* S-2 and 312 spores and their spores treated by L-alanine. As shown in Figure 3, the spores of *B. subtilis* S-2 and 312 appeared to be in a dormant state, and most of the cells showed bright circles under TEM. After treatment with L-alanine, most of the *B. subtilis* S-2 spores turned gray. However, the *B. subtilis* 312 spores remained in the dormant state, and bright cycles did not produce any change in comparison to the spores without L-alanine treatment.

### Comparative Transcriptome Analysis of *B. subtilis* S-2 and 312 in Response to L-Alanine

**Figure 4:**
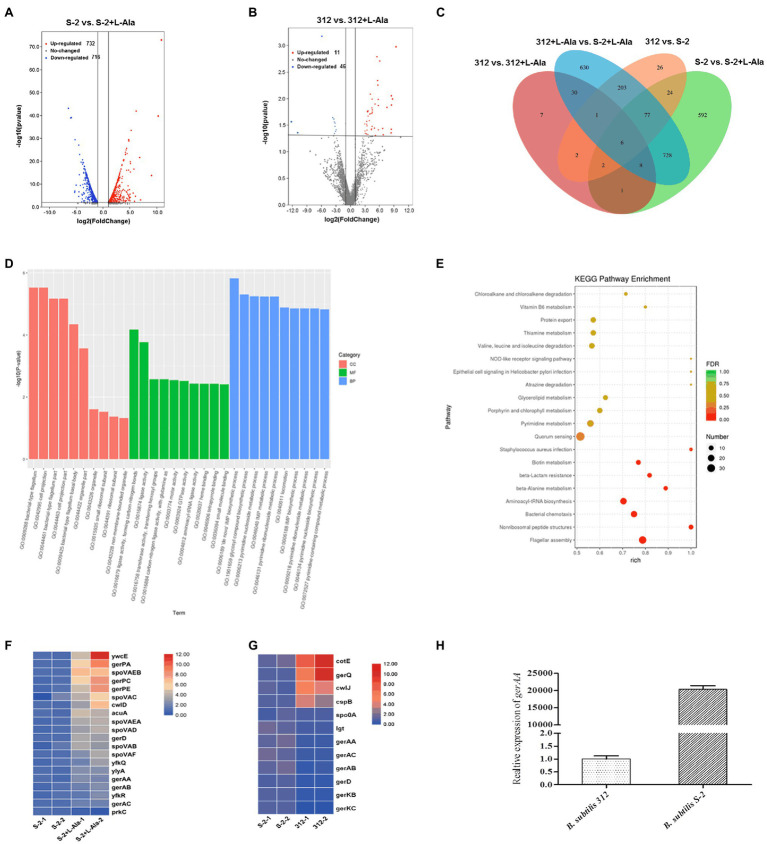
Comparative transcriptome analysis of *B. subtilis* S-2 and 312 strains in response to L-alanine. **(A)** Volcano plot of differently expressed genes (DEGs) between *B. subtilis* S-2 spores and L-alanine pre-treated *B. subtilis* S-2 spores. **(B)** Volcano plot of DEGs in *B. subtilis* 312 spores and L-alanine pre-treated 312 spores. **(C)** Venn diagram of the numbers of DEGs between 4 groups. **(D)** GO analysis of the DEGs between *B. subtilis* S-2 spores and L-alanine pre-treated *B. subtilis* S-2 spores. **(E)** GO analysis of the DEGs between *B. subtilis* 312 spores and L-alanine pre-treated *B. subtilis* 312 spores. **(F)** DEGs enriched in spore germination pathway between *B. subtilis* S-2 spores and L-alanine pre-treated *B. subtilis* S-2 spores. Each row represents one gene, and each column represents one sample; Red indicates high expression, and blue represents low expression. **(G)** DEGs enriched in spore germination pathway between *B. subtilis* S-2 and *B. subtilis* 312. Each row represents one gene, and each column represents one sample; Red indicates high expression, and blue represents low expression. **(H)** The expression level of *gerAA* in *B. subtilis* S-2 and 312 spores.

The differences between the *B. subtilis* S-2 and 312 spores in response to L-alanine germination at the gene level were further studied based on the comparative transcriptome analysis. As seen in Figure 4A, a total of 1,438 genes were significantly changed in *B. subtilis* S-2 after L-alanine treatment (S-2 vs. S-2 + L-alanine), among which 716 genes were up-regulated and 722 genes were down-regulated. However, only 48 genes were up-regulated, and 11 genes were down-regulated in *B. subtilis* 312 after L-alanine treatment (312 vs. 312 + L-alanine; Figure 4B). These results suggest that more genes are involved in the L-alanine response in *B. subtilis* S-2 in comparison to *B. subtilis* 312.

Figure 4C shows a Venn diagram of the number of DEGs among different groups. A total of 341 DEGs were found between *B. subtilis* 312 and *B. subtilis* S-2, most of which were overlapped to the DEGs in the L-alanine-treated *B. subtilis* S-2 vs. L-alanine-treated *B. subtilis* 312 group. Only 17 DEGs were the same between S-2 vs. S-2 + L-alanine group and 312 vs. 312 + L-alanine group. This indicates that there are significant differences in the DEGs responding to L-alanine between the two strains. However, the S-2 vs. S-2 + L-alanine group and the S-2 + L-alanine vs. 312 + L-alanine group shared the largest number (819) of DEGs.

The GO and KEGG pathway enrichment analyses for the DEGs in the S-2 strain before and after L-alanine treatment (S-2 vs. S-2 + L-alanine) were also conducted. The GO functional enrichment analysis found that the DEGs were mainly included in biological processes (Figure 4D). The *De Novo* IMP biosynthetic progress was the most enriched pathway. The secondary enrichment pathways also included flagellum formation, biosynthetic pathways, and enhanced nucleotide metabolism. These results indicate that the main function of L-alanine was to promote the biosynthesis and growth of *B. subtilis* S-2. The KEGG pathway analysis showed that the main enriched pathways of DEGs primarily included quorum sensing, flagellar assembly, and nonribosomal peptide structure (Figure 4E). Figures 4F,G showed the DEGs annotated to spore germination term (GO:0009847) between different groups. It was found that 20 germination-related genes were differentially expressed after L-alanine treatment in *B. subtilis* S-2; only one gene, *spoVAA*, was differentially expressed in *B. subtilis* 312 (data not shown). A total of 12 germination-related genes were differentially expressed in *B. subtilis* S-2 and *B. subtilis* 312, with four up-regulated genes and eight down-regulated genes (Figure 4G). The expression level of the L-alanine receptor gene, *gerAA*, in the two strains was verified by RT-qPCR. As seen in Figure 4H, the relative expression of *gerAA* gene was about 20,000-fold greater in *B. subtilis* S-2 than that in *B. subtilis* 312.

### The Different Effects of *B. subtilis* S-2 and 312 Exerted by L-Alanine on the Inflammatory Cytokines and Intestinal Barrier in IPEC-J2 Cells

**Figure 5:**
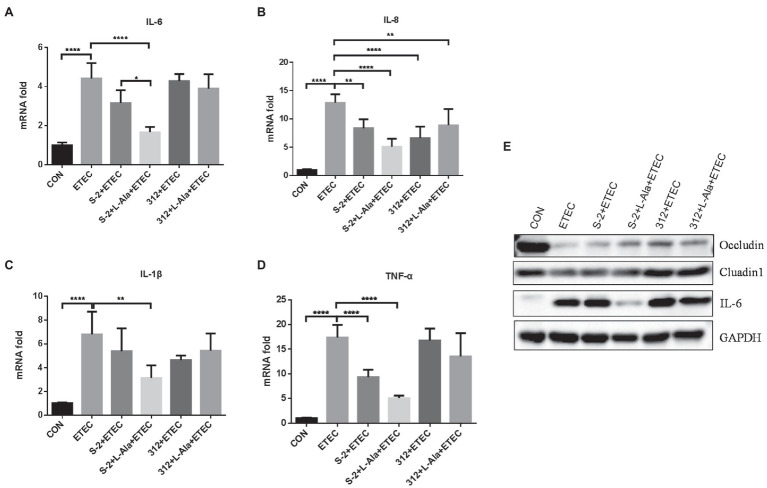
The different effects of L-alanine mediated *B. subtilis* S-2 and 312 strains on inflammatory cytokines and tight junction proteins in IPEC-J2 cells. IPEC-J2 cells were incubated with *B. subtilis* S-2 and 312 spores (10^6^ CFU/ml) with or without L-alanine pre-treated for 16 h. Then the cells were infected with ETEC-K99 (10^6^ CFU/ml) for 12 h. The mRNA expression of IL-6 **(A)**, IL-8 **(B)**, IL-1 β **(C)**, TNF-α **(D)** were detected by RT-qPCR; **(E)** The protein expression of occludin, claudin, and IL-6 were tested by WB. Values are expressed as the mean ± SD from three independent experiments. ^*^p < 0.05; ^**^p < 0.01; ^****^p < 0.0001, as calculated by one-way ANOVA with Tukey’s multiple comparisons test. Groups with no significant difference were not marked.

The protective effect of L-alanine-induced spore germination on the IPEC-J2 cells infected by ETEC K99 was compared. As shown in Figures 5A–D, ETEC K99 infection significantly increased the mRNA expression level of the inflammatory factors, IL-6, IL-8, IL-1 β, and TNF-α, in the IPEC-J2 cells in comparison to the control (*p* < 0.0001). In contrast to the ETEC K99 infection group, the *B. subtilis* S-2 spores decreased the mRNA expression of the four inflammatory cytokines, and the levels of IL-8 and TNF-α were significant (*p* < 0.01 and *p* < 0.001, respectively). The *B. subtilis* 312 spores significantly decreased the mRNA expression level of IL-8 (*p* < 0.0001). Interestingly, the *B. subtilis* S-2 spores treated by L-alanine tended to further reduce the mRNA expression of IL-6, IL-8, IL-1 β, and TNF-α in comparison to the *B. subtilis* S-2 spores alone, and the decrease in the level of IL-6 was significant (*p* < 0.05). However, there was no difference in the effect on inhibition of the inflammatory factors between the L-alanine-treated *B. subtilis* 312 spores and *B. subtilis* 312 spores alone.

The expression of tight junction proteins and IL-6 was further tested by WB, as shown in Figure 5E. It was found that ETEC K99 infection significantly increased the expression of IL-6 and decreased the expression of occludin and cluadin1 compared to the control. Compared to the untreated *B. subtilis* S-2 spores, the L-alanine-induced S-2 spores increased the expression of occludin and decreased the expression of IL-6. *B. subtilis* 312 spores and their L-alanine induced spores both up-regulated the expression of occludin and cluadin1. However, no difference was observed between the *B. subtilis* 312 spores treated with L-alanine and those not treated with L-alanine.

### Promoting Effects of L-Alanine-Mediated Spore Germination on Rat Growth and the Intestinal Barrier

**Figure 6:**
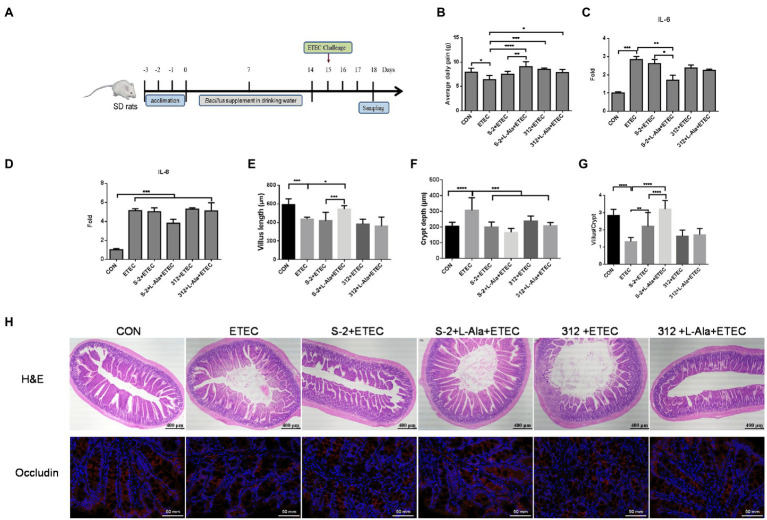
Promoting effects of L-alanine-mediated spore germination on animal growth and intestinal barrier. **(A)** Diagram illustrating the ETEC-challenge model employed in this study. **(B)** Average daily gain of SD rats feeding *B. subtilis* S-2 and 312 spores with or without L-alanine treated from day 15 to day 18. **(C,D)** The mRNA fold of IL-6 and IL-8 in the jejunum of SD rats feeding different spores. **(H)** H&E stain (up line) and immunofluorescence analysis (IFA; bottom) of occludin in the jejunum of SD rats. Occludin was stained as red, and DAPI was stained as blue. **(E–G)** The villus length, crypt depth, and villus length/crypt depth ratio in jejunum of different groups, respectively. Values are expressed as the mean ± SD from at least 6 animals. ^*^p < 0.05; ^**^p < 0.01; ^***^p < 0.001; ^****^p < 0.0001, as calculated by one-way ANOVA with Tukey’s multiple comparisons test. Groups without significant difference were not marked.

The effect of spore germination induced by L-alanine was investigated using a rat challenge model (Figure 6A). Figure 6B shows that the average daily gain (ADG) of the SD rats was significantly decreased after ETEC K99 infection in contrast to the control (*p* < 0.05). Administration of the spores from the untreated *B. subtilis* S-2 and *B. subtilis* 312 or their associated L-alanine-treated spores all significantly increased the ADG of the rats (*p* < 0.05) and the ADG level of the L-alanine-treated *B. subtilis* S-2 spores was the highest (*p* < 0.0001) in comparison to the ETEC K99 groups. The ETEC K99 infection increased the expression of the intestinal cytokines IL-6 and IL-8 (Figures 6C,D). However, administration of the L-alanine-treated *B. subtilis* S-2 spores significantly decreased IL-6 expression in the jejunum compared to the ETEC K99 group and the *B. subtilis* group (*p* < 0.01 and *p* < 0.05, respectively; Figure 6C).

The H&E staining results revealed that ETEC infection caused shortened villi, larger space among the villi, and loss of epithelial cells in the intestinal epithelium of the jejunal structure in the SD rats (Figure 6H). Administration of the L-alanine-treated *B. subtilis* S-2 spores attenuated the ETEC-induced jejunal mucosa lesions and improved the expression of occludin (Figure 6H). The length of the jejunal villi was significantly decreased (*p* < 0.001), and the crypt depth was increased (*p* < 0.0001) in the ETEC group in comparison to the control (Figures 6E,F). The crypt depths were decreased in all four of the *Bacillus* administration groups. However, only the L-alanine-induced *B. subtilis* S-2 spores significantly increased the villi length (*p* < 0.05) and the ratio of villus length to crypt depth (*p* < 0.0001; Figures 6E,G).

### Regulating Effects of L-Alanine-Mediated Spore Germination on Gut Microbiota

**Figure 7:**
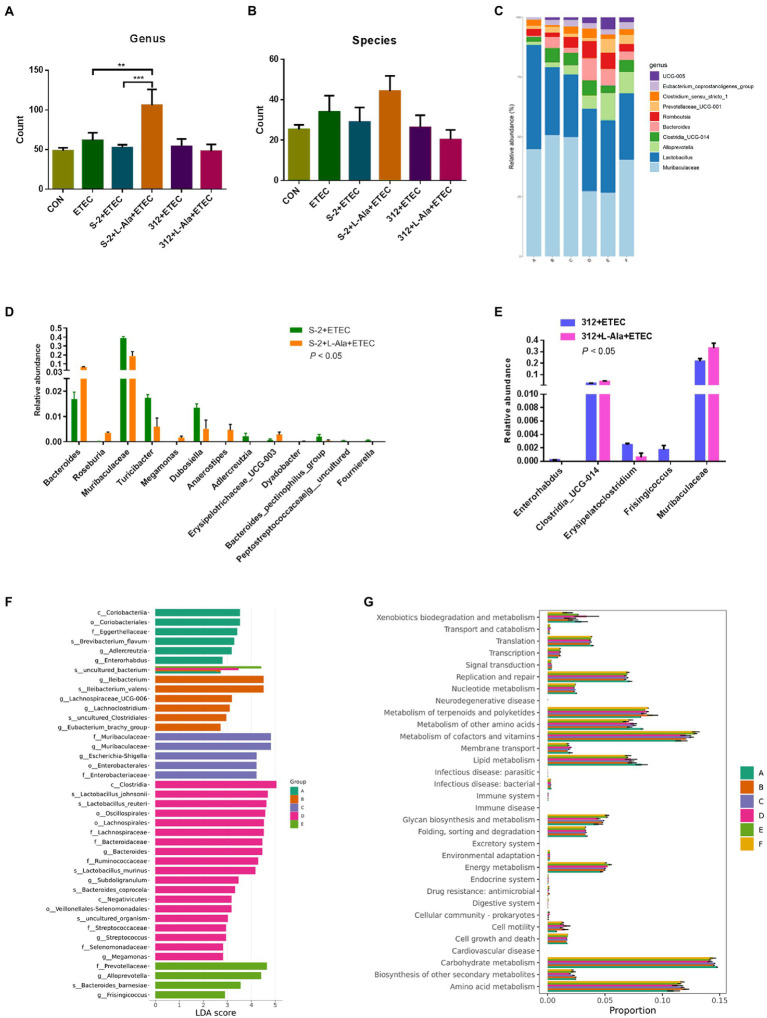
Regulating effects of L-alanine-mediated spore germination on gut microbiota. **(A)** Number of bacteria detected at genus level in different treatments. **(B)** Number of bacteria detected at species level in different treatments. **(C)** Histogram of the top 10 abundant genera in each group. **(D)** The taxa with significant differences (*p* < 0.05) in abundance at genus level between *B. subtilis* S-2 and S-2 + L-alanine groups. **(E)** The taxa with significant differences (*p* < 0.05) in abundance at genus level between 312 and 312 + L-alanine groups. **(F)** Histogram of LDA value distribution. **(G)** KEGG pathway analysis based on the abundance of marker gene sequences in the samples. There are 3 samples in each group. (Group A represents CON, Group B represents ETEC, Group C represents S-2 + ETEC, Group D represents S-2 + L-alanine + ETEC, Group E represents 312 + ETEC, Group F represents 312 + L-alanine + ETEC.) Values are expressed as the mean ± SD. ^**^p < 0.01; ^***^p < 0.001, as calculated by one-way ANOVA with Tukey’s multiple comparisons test.

The microbiome of the rat feces was explored to study the discrepancy resulting from the administration of spores treated by L-alanine. As seen in Figures 7A,B, the L-alanine-treated *B. subtilis* S-2 spores had the highest number of bacterial genus and species, and the increase in the genus level was significant (*p* < 0.01). Additionally, the L-alanine-treated *B. subtilis* S-2 spores tended to promote the abundance of *Lactobacillus*, *Bacteroides*, *Romboutsia,* and UCG-005 in the rat feces (Figure 7C).

The abundance of the taxa at the genus level was also analyzed. A total of 13 genera were found to have significant changes in taxa abundance between the S-2 and S-2 + L-alanine groups (*p* < 0.05; Figure 7D). In contrast, the taxa abundance of only 5 genera was significantly changed between the 312 and 312 + L-alanine groups (*p* < 0.05; Figure 7E). A total of 10 taxa were found to have significant differences in abundance at the genus level between the ETEC and S-2 + ETEC groups (Supplementary Table 1), and 20 taxa were found to have a significant difference in abundance at the genus level between the ETEC and S-2 + ETEC groups (Supplementary Table 2).

Next, LDA was performed to identify the biomarkers with a significant difference in taxa abundance in each group (Figure 7F). It was found that the L-alanine-pretreated *B. subtilis* S-2 had the largest number of species with a significantly different abundance in comparison to the other groups. The functional analysis of the samples showed that the gene functions of the six groups were mostly enriched in carbohydrate metabolism, amino acid metabolites, metabolism of cofactors, and the vitamin pathways (Figure 7G).

## Discussion

The massive use of antibiotics leads to the emergence of antimicrobial resistance, which poses a great threat to human health and animal production. Therefore, it is important to find suitable alternatives to antibiotics. Studies have shown that *Bacillus* species have been used as probiotics in medical supplements and livestock feeds for more than 60 years, and scientific interest has increased significantly in the last two decades (Hong et al., 2005; Cutting, 2011; Piewngam et al., 2018). The most attractive advantages of using *Bacillus* species as probiotics are their high stability and their significant capacity for metabolic activity, including the production of various enzymes, antimicrobial metabolites, pentapeptide, and lipopeptides, such as fengycin, coagulin, and surfactin (Piewngam et al., 2018). However, metabolic activity is highly associated with the germination process, which is dependent on the strain itself and the intestinal environmental conditions (Bernardeau et al., 2017). The complexity and dynamics in the GIT of *Bacillus* probiotics are largely unknown since the *Bacillus* species are ingested in the form of spores. Thus, it is crucial to explore the relationship between *Bacillus* strain-related germination and the influence of their protective effect in the gut.

In the present study, we confirmed the strain-specific and germinant-related properties of spores in response to germination. As seen in Figures 1A–D, not all spores from the *Bacillus* species are responsible for the germination triggered by nutrients, including L-alanine. As a sole nutrient-germinant, L-alanine most often triggers the germination of spore-formers (Moir and Smith, 1990; Lovdal et al., 2012). The spore germination assay of the 15 *Bacillus* strains involved in this study also supports the view that L-alanine is an important nutrient-germinant for different *Bacillus* species.

The results shown in Figure 2 further indicate that L-alanine-triggered gemination promotes the proliferation of spores in a restricted medium, which is similar to what occurs in the intestinal environment. When dormant spores sense an environment with specific nutrients conducive to vegetative growth, they can rapidly return to active growth through germination followed by outgrowth (Moir and Cooper, 2015; Christie and Setlow, 2020). The growth of vegetative *B. subtilis* under anaerobe conditions has been verified from the early report (Nakano and Zuber, 1998). Therefore, in the intestinal environments, the presence of potential nutrient agents, such as amino acids, sugars, ribosides, and some inorganic salts, could be provided to promote spore germination in the gut. Previous reports have verified that ingested *Bacillus* spores can germinate in varying proportions and have whole life cycles in the GIT of mice, pigs, and chickens (Tam et al., 2006; Bernardeau et al., 2017). However, the persistence of *Bacillus* strains in the GIT is species- and strain-specific. As seen in Figure 2A, the L-alanine growth-promoting effect was only observed in the L-alanine-induced *B. subtilis* S-2 strain. This result indicates that the germination-specific strain contributes to the germination and proliferation of spores, which enhances the persistence of *Bacillus* strains in the GIT.

The differential morphological changes of *B. subtilis* S-2 and *B. subtilis* 312 after L-alanine treatment were further verified by TEM. Because of the thick, multilayered structure of spores with low water content, they were highly refractive and difficult to stain. The spores often showed bright circles under TEM. However, after the release of DPA and the water uptake by the spores triggered by the germinants, the germinating spores changed from the transparent phase to the gray phase. The germination process could also be captured through phase contrast microscopy, in which the germinated and dormant spores appeared dark and white/bright, respectively (Lovdal et al., 2012; Amon et al., 2020). In the present study, TEM also clearly showed that *B. subtilis* S-2 germinated with L-alanine, and there was no apparent increase in the number of gray spores of *B. subtilis* 312 supplemented with L-alanine or the negative controls. The results of the differential TEM images of the two strains responding to L-alanine were also consistent with the results of the germination assay and growth assay.

The germination triggered by the nutrient germinants are closely associated with the specific receptors located in the spore’s inner membrane, resulting in the release of DPA from the spore core and subsequent cortex degradation (Chirakkal et al., 2002; Christie and Setlow, 2020). Different *Bacillus* strains have different germinant receptors that recognize specific nutrient germinants. The comparative transcriptome results showed that the DEGs of *B. subtilis* S-2 responding to L-alanine was far greater than the DEGs of *B. subtilis* 312, and the spore germination-related genes (*ywcE*, *gerPA*, *spoVAEB*, *gerPC*, *gerPE*, *spoVAC*, *cwlD*, *acuA*, etc.; Figure 4). Moreover, the DEGs were principally enriched in metabolism, cellular processes, and biosynthetic pathways. To be specific, *ywcE* is important for proper spore morphogenesis (Real et al., 2005), and *GerPA*, *GerPC*, and *GerPE* are involved in the establishment of normal spore coat structure and permeability, which allows the germinants to access their receptors (Casula and Cutting, 2002; Chirakkal et al., 2002; Moir et al., 2002; Ghosh et al., 2018). The up-regulated *acuA* gene helped mediate the post-translational regulation during the 80 min to 100 min of spore germination and into the outgrowth phase (Gardner and Escalante-Semerena, 2008). The L-alanine-induced germinant receptor in *B. subtilis* is encoded by the *gerA* operon, *gerAA*, *gerAB*, and *gerAC* (Mongkolthanaruk et al., 2011). Consequently, the expression of the germinant receptors is much higher in the *B. subtilis* S-2 strain than the *B. subtilis* 312 (Figure 4H). These findings confirm that differences in gene expression between the *B. subtilis* S-2 and 312 strains are due to differences in the germination and growth responses to L-alanine. The higher expression of the germination receptors promoted the germination response in the *Bacillus* strains.

The anti-inflammatory and immunomodulatory functions of probiotic *B. subtilis* have been confirmed in other studies (Du et al., 2018; Gong et al., 2018; Luise et al., 2019). However, probiotic activities are often strain-related even in the same reports (Hu et al., 2018; Rhayat et al., 2019). When considering the specific germination of spores, in the present study, the effect of L-alanine-induced germination of *B. subtilis* S-2 and *B. subtilis* 312 spores on the protective activities was intensively investigated in ETEC challenged intestinal epithelial cells and SD rats *in vitro* and *in vivo*, respectively. The ETEC K99 challenge model was successful in both the cell model and animal model since the inflammatory cytokines were significantly up-regulated after the challenge (Figures 5, 6). Both the *B. subtilis* S-2 and *B. subtilis* 312 spores shared some anti-inflammatory activity by significantly decreasing the expression of some of the inflammatory cytokines at the IPEC-J2 cells (Figure 5). Moreover, after the challenge, the rat growth performance was improved by administration of the spores from the two strains (Figure 6). The beneficial activities of the two spores might be associated with the immune effects of the spores, which was not strain-specific since the spores and vegetative cells exhibit differential functional properties (Foligne et al., 2012). However, from a living *Bacillus* perspective, the probiotic effect is the result of the complementary action of vegetative cells and spores (Bernardeau et al., 2017). The bacterial spores are metabolically dormant, and vegetative cells provide more health benefits because of their metabolic activity and growth potential. Importantly, our study showed that the spores of *B. subtilis* S-2 triggered by L-alanine can more effectively inhibit the expression of inflammatory factors, promote the expression of tight junction proteins, and help repair intestinal barrier injury. The probiotic function was significantly increased after L-alanine induced the alteration of *Bacillus* germination both in the IPEC-J2 cell model and in the rat model. To the best of our knowledge, this is the first report to show that L-alanine-induced *Bacillus* spore germination can improve the protective activity against ETEC K99-induced intestine injury.

Probiotic *Bacillus* species are beneficial for modulating the intestinal microflora and micro-ecological balance (Motlagh et al., 2012; Wang et al., 2017; Luo et al., 2020). In the present study, the increased intestinal microbiota barrier by the L-alanine-induced spores was further confirmed in the SD rats (Figure 7). The diversity of fecal microbiota was only significantly enhanced in the L-alanine-induced *B. subtilis* spores group (*p* < 0.01). Thus, an environment with more highly diverse gut microbiota could be more stable and healthier than an environment with gut microbiota that is less diverse (Li et al., 2018). The increased abundance of *Lactobacillus*, *Bacteroides*, *Romboutsia*, UCG-005 in the rats ingesting the L-alanine-induced spores contributed to creating a better intestinal environment. The increased microbiota function of the spores induced by L-alanine was in accordance with the results in the anti-inflammatory and growth-promoting effect shown in Figures 5, 6. The improved intestinal microbiota by *Bacillus* administration might be caused by the metabolic activity and biological oxygen-capturing potential (Yu et al., 2019), which are both closely related to the germination and proliferation of spores in the gut. Therefore, the results of the intestinal microbiota analyses also support the view that the germination of spores initiated by L-alanine could result in an increased probiotic effect.

In summary, we first described that L-alanine-induced *Bacillus* germination increased the protective effects by alleviating ETEC K99-induced intestine injury and enhancing the intestinal microbiota barrier. We propose that L-alanine works well as a probiotic *Bacillus* adjuvant based on the strain’s germination potential in improving intestinal health. It also provides a solution for the accurate screening of *Bacillus* probiotics and the practical and accurate regulation of their use as antibiotic alternatives in animal production.

## Data Availability Statement

The datasets presented in this study can be found in online repositories. The names of the repository/repositories and accession number(s) can be found in the article/Supplementary Material.

## Ethics Statement

The animal study was reviewed and approved by the Ethical Committee of South-Central University for Nationalities.

## Author Contributions

XG conceived and designed the experiments and revised the manuscript. SL, XL, LZ, YF, and MX performed the experiments. SL and XG analyzed the data and made the figures. SL wrote the original draft. All authors reviewed and approved the final manuscript.

## Funding

This study was supported by the National Natural Science Foundation of China (Nos. 32072767 and 31672455), the Fundamental Research Funds for Central Universities South-Central University for Nationalities (No. CZZ21011), and the Fundamental Research Funds for Health Commission of Hubei Province (ZY2021M061).

## Conflict of Interest

The authors declare that the research was conducted in the absence of any commercial or financial relationships that could be construed as a potential conflict of interest.

## Publisher’s Note

All claims expressed in this article are solely those of the authors and do not necessarily represent those of their affiliated organizations, or those of the publisher, the editors and the reviewers. Any product that may be evaluated in this article, or claim that may be made by its manufacturer, is not guaranteed or endorsed by the publisher.

## Footnotes

^1^ https://www.ncbi.nlm.nih.gov/assembly/GCF_000009045.1/

^2^ http://bowtie-bio.sourceforge.net/index.shtml

## Supplementary Material

Supplementary Figure 1 Phylogenetic tree based on the 16S ribosomal RNA sequences from 26 *Bacillus* strains. The tree was constructed using the neighbor-joining method with MEGA 5.0. Branch quality was assessed by the bootstrap test using 1,000 replications.
