# Supplementary material for: Nutrient L-Alanine-Induced Germination of Bacillus Improves Proliferation of Spores and Exerts Probiotic Effects in vitro and in vivo
Source: Front Microbiol. 2021 Dec 2;12:796158. doi: 10.3389/fmicb.2021.796158 (PMC8675871; doi:10.3389/fmicb.2021.796158)
Supplement: Supplementary file 2 [file Table_1.DOCX]

**Supplementary Tables**

Supplementary Table 1. The taxa with significant differences in abundance at genus level between ETEC and S-2+ETEC groups

| Taxa | *P* value | UP/DOWN |
| --- | --- | --- |
| Firmicutes\|c__Bacilli\|o__Erysipelotrichales\|f__Erysipelotrichaceae\|g__Turicibacter | 0.00199 | UP |
| Firmicutes\|c__Clostridia\|o__Lachnospirales\|f__Lachnospiraceae\|g__Lachnoclostridium | 0.01185 | DOWN |
| Firmicutes\|c__Clostridia\|o__Peptostreptococcales-Tissierellales\|f__Anaerovoracaceae\|g__Eubacterium_brachy_group | 0.0128 | DOWN |
| Firmicutes\|c__Clostridia\|o__Peptostreptococcales-Tissierellales\|f__Anaerovoracaceae\|g__Eubacterium_nodatum_group | 0.01375 | DOWN |
| Firmicutes\|c__Clostridia\|o__Oscillospirales\|f__Ruminococcaceae\|g__Incertae_Sedis | 0.01582 | DOWN |
| Actinobacteriota\|c__Actinobacteria\|o__Bifidobacteriales\|f__Bifidobacteriaceae\|g__Bifidobacterium | 0.01765 | UP |
| Firmicutes\|c__Clostridia\|o__Monoglobales\|f__Monoglobaceae\|g__Monoglobus | 0.0186 | DOWN |
| Bacteroidota\|c__Bacteroidia\|o__Bacteroidales\|f__Bacteroidaceae\|g__Bacteroides | 0.01956 | DOWN |
| Firmicutes\|c__Clostridia\|o__Clostridiales\|f__Clostridiaceae\|g__Clostridium_sensu_stricto_1 | 0.02726 | UP |
| Bacteroidota\|c__Bacteroidia\|o__Bacteroidales\|f__Muribaculaceae\|g__Muribaculaceae | 0.03108 | UP |

Supplementary Table 2. The taxa with significant differences in abundance at genus level between ETEC and S-2+L-alanine+ETEC groups

| Taxa | *P* value | UP/DOWN |
| --- | --- | --- |
| Firmicutes\|c__Clostridia\|o__Lachnospirales\|f__Lachnospiraceae\|g__Roseburia | 0.00115 | UP |
| Bacteroidota\|c__Bacteroidia\|o__Bacteroidales\|f__Bacteroidaceae\|g__Bacteroides | 0.00167 | UP |
| Firmicutes\|c__Clostridia\|o__Peptostreptococcales-Tissierellales\|f__Anaerovoracaceae\|g__Eubacterium_brachy_group | 0.00219 | DOWN |
| Firmicutes\|c__Clostridia\|o__Peptostreptococcales-Tissierellales\|f__Anaerovoracaceae\|g__Eubacterium_nodatum_group | 0.00378 | DOWN |
| Firmicutes\|c__Clostridia\|o__Clostridiales\|f__Clostridiaceae\|g__Clostridium_sensu_stricto_1 | 0.01062 | UP |
| Bacteroidota\|c__Bacteroidia\|o__Bacteroidales\|f__Muribaculaceae\|g__Muribaculaceae | 0.01114 | DOWN |
| Bacteroidota\|c__Bacteroidia\|o__Bacteroidales\|f__Prevotellaceae\|g__Prevotellaceae_UCG-001 | 0.01166 | DOWN |
| Firmicutes\|c__Clostridia\|o__Oscillospirales\|f__Ruminococcaceae\|g__Incertae_Sedis | 0.01271 | DOWN |
| Firmicutes\|c__Bacilli\|o__Lactobacillales\|f__Streptococcaceae\|g__Streptococcus | 0.01436 | UP |
| Actinobacteriota\|c__Coriobacteriia\|o__Coriobacteriales\|f__Eggerthellaceae\|g__Adlercreutzia | 0.01488 | DOWN |
| Firmicutes\|c__Negativicutes\|o__Veillonellales-Selenomonadales\|f__Selenomonadaceae\|g__Megamonas | 0.0252 | UP |
| Firmicutes\|c__Clostridia\|o__Christensenellales\|f__Christensenellaceae\|g__Christensenellaceae_R-7_group | 0.03051 | DOWN |
| Actinobacteriota\|c__Coriobacteriia\|o__Coriobacteriales\|f__Eggerthellaceae\|g__Enterorhabdus | 0.03275 | DOWN |
| Bacteroidota\|c__Bacteroidia\|o__Bacteroidales\|f__Prevotellaceae\|g__Alloprevotella | 0.03571 | UP |
| Firmicutes\|c__Clostridia\|o__Lachnospirales\|f__Lachnospiraceae\|g__Anaerostipes | 0.03623 | UP |
| Bacteroidota\|c__Bacteroidia\|o__Bacteroidales\|f__Tannerellaceae\|g__Parabacteroides | 0.03844 | DOWN |
| Firmicutes\|c__Clostridia\|o__Peptostreptococcales-Tissierellales\|f__Peptostreptococcaceae\|g__Romboutsia | 0.03951 | UP |
| Bacteroidota\|c__Bacteroidia\|o__Bacteroidales\|f__Prevotellaceae\|g__Prevotella | 0.04058 | UP |
| Bacteroidota\|c__Bacteroidia\|o__Cytophagales\|f__Spirosomaceae\|g__Dyadobacter | 0.04221 | UP |
| Actinobacteriota\|c__Coriobacteriia\|o__Coriobacteriales\|f__Eggerthellaceae\|g__DNF00809 | 0.04442 | DOWN |
